# The International Congress of Gynæcology and Obstetrics

**Published:** 1900-10-20

**Authors:** 


### THE INTERNATIONAL CONGRESS OF GYNÆCOLOGY AND OBSTETRICS.

Dr. Jacobs, of Brussels, the permanent secretary of the International Congress of Gynæcology and Obstetrics, has written a letter to the secretary of the British Gynæcological Society thanking it for its efforts in regard to the proposal to hold the next session of the Congress in London in the year 1902, and expressing a hope that at some future time the Congress may meet here. By postponing the meeting for some years it is to be hoped that when it does take place the Congress will receive a hearty welcome, not from one or other society or group of practitioners but from the gynæcologists of England.

